# Reported xylazine exposure highly associated with overdose outcomes in a rapid community assessment among people who inject drugs in Baltimore

**DOI:** 10.1186/s12954-024-00940-z

**Published:** 2024-01-22

**Authors:** Danielle German, Becky Genberg, Olivia Sugarman, Brendon Saloner, Anne Sawyer, Jennifer L. Glick, Molly Gribbin, Colin Flynn

**Affiliations:** 1 Department of Health, Behavior, and Society, Bloomberg School of Public Health, Johns Hopkins University, 624 N. Broadway, Baltimore, MD 21205 USA; 2 Department of Epidemiology, Bloomberg School of Public Health, Johns Hopkins University, Baltimore, MD USA; 3 Department of Health, Policy and Management, Bloomberg School of Public Health, Johns Hopkins University, Baltimore, MD USA; 4 Maryland Department of Health, Baltimore, MD USA

**Keywords:** Xylazine, Overdose, People who inject drugs, PWID, Wound

## Abstract

**Background:**

Addressing xylazine harms are now a critical harm reduction priority, but relatively little epidemiological information exists to determine prevalence, magnitude, and correlates of xylazine use or related outcomes.

**Methods:**

We conducted a rapid behavioral survey among people who inject drugs (*n* = 96) in Baltimore November–December 2022. Using a novel indicator of self-reported presumed xylazine effects, we examined prevalence and sociodemographic correlates of past year presumed xylazine effects and association with overdose and wound-related outcomes. Chi-square and descriptive statistics were used to examine bivariate associations overall and separately for those who reported xylazine by name and by reported fentanyl use frequency.

**Results:**

Almost two-thirds (61.5%) reported experiencing xylazine effects. There were no differences by socio-demographics, but xylazine effects were more commonly reported among those who reported injecting alone (66% vs 38%%, *p* < 0.007) and daily fentanyl use (47% vs 24% *p* < 0.003). Those reporting xylazine exposure was three times as likely to report overdose (32% vs 11%, *p* < 0.03) and twice as likely to have used naloxone (78% vs 46%, *p* < 0.003). They also more commonly reported knowing someone who died of an overdose (92% vs 76%, *p* < 0.09) and to report an abscess requiring medical attention (36% vs 19%, *p* < 0.80). These associations were higher among respondents who specifically named xylazine and those who used fentanyl more frequently, but fentanyl frequency did not fully explain the heightened associations with xylazine effects.

**Conclusions:**

This study provides insight into the scope of xylazine exposure and associated health concerns among community-based PWID and suggests measures that may be instrumental for urgently needed research.

## Introduction

Xylazine is a non-opioid large animal sedative increasingly combined with opioids, especially fentanyl. Recent national alerts have highlighted serious risk for overdose and skin wounds for people using substances that include xylazine [1, 2]. Fentanyl combined with xylazine is now designated a U.S. ‘emerging threat’ based on its growing role in overdose deaths and rapid geographic distribution nationwide [3].

There are major epidemiological questions about xylazine and its relationship to health risks. Overdose fatality data show xylazine-related death distribution across the US [4] but is inherently limited to decedents. It also provides no information about xylazine implications in non-fatal overdoses for which survival may be dependent on available revival resources. Xylazine prevalence and population risk factors have not been quantified and data on related wounds are extremely limited.

While some people who inject drugs (PWID) may intentionally use xylazine, it is often added without their knowledge [5, 6]. In the absence of universal drug testing, it is important to identify indicators of presumed xylazine presence for use in harm reduction communication and survey research. This study was designed as a rapid assessment to: (1) examine frequency of reported exposure to xylazine effects and by name among active PWID using two novel measures, (2) identify whether reported exposure varied by sociodemographic or drug use, and (3) understand relationships between reported xylazine exposure and health-related outcomes.

## Methods

The study uses data from the Baltimore 2022 PWID cycle of National HIV Behavioral Surveillance [7]. Participants were recruited using respondent-driven sampling (RDS) [8] November–December 2022. Eligibility required a valid study coupon, 18+ age, Baltimore–Columbia–Towson residence, and past year drug injection. After informed consent procedures, participants completed an anonymous socio-behavioral survey with common core questions about socio-demographics, injection, overdose, and services. Remuneration was $50 for survey participation, $25 for optional HIV testing, and $10 each for up to 5 referred participants.

Participants also completed locally specific questions informed by formative research involving professional and community key informant interviews as well as focus groups with PWID conducted in Spring 2022. Relatively few respondents mentioned xylazine by name, although there had been some news media in neighboring counties about xylazine as a new concern in the drug supply and some preliminary alerts to harm reduction providers about heightened overdose potential. A few providers reported hearing about ‘tranq’ and xylazine from PWID, as has been reported elsewhere [9], especially in areas more proximate to Philadelphia. Among PWID, there was some awareness of the emerging possible presence of an “animal tranquilizer” in packaged fentanyl associated with a specific set of experiential characteristics consistent with xylazine’s pharmacological effects [10, 11]; specifically, a 20–30 min “very heavy” nod in which the person may not be able to be roused even though they are breathing and dry mouth, which was later clarified as extreme dry mouth. Several providers described concerns about terrible wounds and shifts in withdrawal symptoms with speculation about the drug supply, but most discussions of xylazine were about overdose. The study team developed survey questions to assess possible xylazine exposure based on the formative research, consulting again with key informants familiar with xylazine to finalize wording. The first item: “In the past 12 months, have you used drugs that were cut with Xylazine, sometimes known as tranq, tranq dope, or animal tranquilizer?” asked about xylazine by name, and the second item: “In the past 12 months, after you injected drugs, did you experience a very heavy nod that lasted 20 min or more and extreme dry mouth?” asked about a specific type of high that local experts associated with xylazine exposure.

The study was determined to be non-research by Johns Hopkins Bloomberg School of Public Health and Maryland Department of Health Institutional Review Boards.

We examined presumed xylazine effects (yes/no) in the presence of key sociodemographic covariates and related to drug type, frequency, and context (e.g., use alone, shooting gallery, syringe service program (SSP)). Outcome variables were past year overdose, naloxone, witnessed overdose, and abscess or soft tissue wound requiring medical attention.

Chi-square tests were used for comparisons by reported xylazine effects and then, separately among those reporting xylazine by name compared to those who did not. Separate bivariate analyses were conducted by fentanyl frequency given known associations.

## Results

A total of 96 participants were included. The study population was majority male (71%), older than 45 years (76%), heterosexual (90%), non-Hispanic Black (83%), Baltimore City residents (94%), and reported daily injection (72%). Approximately half reported past year drug or alcohol treatment (55%), injecting in a shooting gallery (47%), and injecting alone (55%), and 70% received syringes from an SSP. Participants most commonly reported daily or more heroin injection (64%), followed by daily or more speedball injection (39%), and less than daily cocaine injection (29%).

Overall, 61% reported experiencing presumed xylazine effects. Of these, 49% reported xylazine exposure by name. Approximately 6% reported xylazine by name but did not report xylazine effects. Presumed xylazine effects did not differ by most sociodemographic and drug use variables (Table 1). Those who reported xylazine effects more commonly reported injecting alone (66% vs 38%, *p* < 0.007) and daily fentanyl use (47% vs 24%, *p* < 0.003).

**Table 1:** Descriptive and health-related characteristics of participants reporting presumed xylazine effects among people who inject drugs in Baltimore (*n* = 96)

	Total	No	Yes	Chi-squared *p*-value
	*N* = 96	*N* = 37	*N* = 59	
Gender: Female	29% (28)	27% (10)	31% (18)	0.71
Age				0.65
< 45	24% (23)	19% (7)	27% (16)	
45–54	31% (30)	32% (12)	31% (18)	
55+	45% (43)	49% (18)	42% (25)	
Race/ethnicity				0.83
Non-Hispanic Black	83% (80)	81% (30)	85% (50)	
Non-Hispanic White	10% (10)	11% (12)	10% (6)	
Other	6% (6)	8% (3)	5% (3)	
Residence: Baltimore City	94% (90)	97% (36)	92% (54)	0.26
Inject daily	72% (69)	62% (23)	78% (46)	0.094
Sexual orientation: Homosexual or Bisexual	10% (10)	8% (3)	12% (7)	0.56
Treatment	55% (53)	65% (24)	49% (29)	0.13
Inject in shooting gallery	47% (45)	41% (15)	51% (30)	0.32
Inject alone	55% (53)	38% (14)	66% (39)	0.007
Syringe service program	70% (67)	65% (24)	73% (43)	0.41
Inject cocaine				0.67
Never	54% (52)	59% (22)	51% (30)	
Less than daily	29% (28)	24% (9)	32% (19)	
Daily or more	17% (16)	16% (6)	17% (10)	
Inject heroin				0.28
Never	11% (11)	16% (6)	8% (5)	
Less than daily	25% (24)	30% (11)	22% (13)	
Daily or more	64% (61)	54% (20)	69% (41)	
Inject speedball				0.069
Never	26% (25)	30% (11)	24% (14)	
Less than daily	35% (34)	46% (17)	29% (17)	
Daily or more	39% (37)	24% (9)	47% (28)	
Fentanyl				0.003
Never	18% (17)	35% (13)	7% (12)	
Less than daily	43% (41)	41% (15)	44% (26)	
Daily or more	39% (37)	24% (9)	47% (28)	
Overdosed in past year	24% (23)	11% (12)	32% (19)	0.030
Used Narcan in past year	41% (39)	22% (8)	53% (31)	0.003
Witnessed overdose in past year	66% (63)	46% (17)	78% (46)	0.001
Knows someone who died of overdose in past year	85% (82)	76% (28)	92% (54)	0.091
Abscess requiring medical attention in past year	29% (28)	19% (7)	36% (21)	0.080

Twenty-four percent of the sample reported overdose in the past year, 66% had witnessed overdose, and 41% had used naloxone (Table 1). Past year overdose was three times higher among those reporting xylazine effects compared to those who did not (32% vs 11%, *p* < 0.030). This group was also more likely to have witnessed overdose (78% vs 46%, *p* < 0.001) and more than twice as likely to have used naloxone in the past year (53% vs 22%, *p* < 0.003). Overall, eighty-five percent knew at least one person who had died of overdose in the past year; half knew at least 4 people; and 27% knew seven or more people who had died of an overdose in the past year. Among those reporting xylazine effects, 92% knew someone who had died of overdose in the past year; compared to 76% among those who did not report effects. Approximately one-third had an abscess or soft tissue wound requiring medical attention in the past year. This was more common among those reporting xylazine effects compared to those who did not (36% vs 19%, *p* < 0.080).

Among those who did not report xylazine by name, all outcomes remained elevated among those reporting presumed xylazine effects, except knowing someone who died of overdose (Table 2). Among those who reported xylazine by name, reporting presumed xylazine effects did not change the association with reported overdose, but naloxone use, overdose witness, and abscesses were further elevated among those reporting xylazine effects; and everyone knew someone who had died of an overdose.

**Table 2:** Proportion of PWID reporting overdose outcomes stratified by reported presumed xylazine effects

Past 12-month outcomes		Overdose experience		Used Narcan		Witnessed overdose		Knows someone who died overdose		Abscess/wound requiring medical attention	
		No %	Yes %	No %	Yes %	No %	Yes %	No %	Yes %	No %	Yes %
Xylazine effect	No (*n* = 37)	44	17	51	21	61	27	67	34	44	25
	Yes (*n* = 59)	56	83	49	79	39	73	33	66	56	75
Xylazine by name	Xylazine effect	No %	Yes %	No %	Yes %	No %	Yes %	No %	Yes %	No %	Yes %
No (*n* = 57)	No (*n* = 29)	56	20	58	35	63	40	67	47	55	38
	Yes (*n* = 28)	43	80	43	65	37	60	33	53	45	62
Yes (*n* = 34)	No (*n* = 6)	17	20	33	5	50	11	0	18	21	13
	Yes (*n* = 28)	83	80	67	95	50	89	0	82	79	87
Fentanyl frequency	Xylazine effect	No %	Yes %	No %	Yes %	No %	Yes %	No %	Yes %	No %	Yes %
None (*n* = 17)	No (*n* = 13)	80	50	80	50	85	50	100	71	0	76
	Yes (*n* = 4)	20	50	20	50	15	50	0	28	0	24
Less than daily (*n* = 41)	No (*n* = 15)	42	20	52	20	50	32	75	32	34	38
	Yes (*n* = 26)	58	80	48	80	50	68	25	68	64	62
Daily (*n* = 37)	No (*n* = 9)	31	9	30	18	44	18	60	19	32	13
	Yes (*n* = 28)	69	91	70	82	56	82	40	81	68	87

Likelihood of each overdose outcome increased with more frequent fentanyl use and each outcome remained notably elevated among those reporting presumed xylazine effects compared to those who did not. The majority of those reporting an abscess requiring medical attention reported fentanyl use and xylazine effects.

## Discussion

Current xylazine estimates rely primarily on data with narrow transferability to community-based populations, which are limited in their ability to determine risk factors and cannot determine patterns of variation among active PWID that could inform interventions. This study shows that a substantial proportion of Baltimore PWID within a community sample experienced presumed xylazine exposure based on reported effects, and many reported exposure by name. There was high concordance between the effect definition agreed upon by key informants and the separate item specifying xylazine by name. Self-reported survey items play a critical role in understanding drug use patterns, trends, risks, and harm reduction needs [12]. A recent review highlighted the urgent need for research insights that can help to inform xylazine response; the dearth of xylazine research in humans; the need to understand xylazine prevalence, trends, and health impacts at a population level; and the importance of meaningful inclusion of people who use drugs in research [13]. This study shows that a set of new measures developed with harm reduction partners can be informative about the extent of reported physical effects consistent with xylazine, perceived exposure to xylazine, and experiences of overdose and wounds among those reporting such exposure. With further evaluation, these measures may be able to help fill important gaps in our understandings of prevalence, risk and protective factors, and implications of xylazine exposure.

Lack of variation in presumed xylazine exposure by most demographic and drug use patterns suggests broad population reach. Mortality data have shown xylazine-involved overdose deaths to be higher among males, non-Hispanic white individuals, and 25–44 year olds [4, 14]. The demographic differences observed in this study could reflect real differences in xylazine exposure patterns, or more likely suggest that those experiencing fatal xylazine-involved overdoses differ from the full population of people exposed. The association between xylazine and fentanyl is consistent with drug checking [15] and overdose fatality indicators [10, 16, 17]. Observed differences in overdose and wound prevalence across fentanyl frequency groups may reflect increased frequency of xylazine exposure.

The substantially elevated experience of overdose is consistent with fatality report indications and could reflect injection practices. For example, people in the xylazine group were much more likely to report the independent risk factor of solitary use. People who recognize xylazine by name may be more knowledgeable about the drug supply and associated risks, either through their networks or community awareness and may be helping disseminate risk reduction insights. Further information is needed to understand mechanisms underlying xylazine-related overdose mortality to inform prevention and response. Meanwhile, many are developing new protocols for overdose response, and there are increasing calls for emergency responder retraining to better respond to the respiratory depressant and sedative effects of xylazine [9]. A recent study showed that xylazine-involved overdose death circumstances are relatively similar to those without xylazine, although fewer have evidence of no pulse upon first responder arrival [18]. Another found xylazine presence to be protective against more severe cardiovascular consequences among those admitted to emergency departments for overdose [19]—although many overdose experiences do not result in emergency department admission.

Almost forty percent of those reporting xylazine effects reported an abscess or wound requiring medical attention in the past year. This is consistent with broad indications that xylazine can create skin lesions which if left untreated lead to severe wounds [20, 21]. PWID peer knowledge sharing has always been instrumental in the face of health risks and structural and healthcare stigma [22, 23]. Ensuring that PWID have access to accurate information and non-judgemental healthcare will be critical for staying ahead of xylazine health consequences. Recent xylazine test strip availability is helpful, although experiences with fentanyl test strips suggest there will quickly be a need to understand not just whether xylazine is present but in what concentration—and whether safer thresholds exist.

Further study will be needed. The sample size limits the analysis that can be done and will strongly benefit from future research with more robust samples. Data were analyzed without RDS weights due to equilibrium constraints. The survey items have also not been evaluated against a biologic indicator of exposure, which would help to evaluate construct validity for use in future studies. This study is not able to determine why some PWID report xylazine exposure, and it is critical that further research helps to distinguish awareness of the drug name from awareness of and perceptions of xylazine’s physical effects. It is also possible that results are not generalizable outside of Baltimore. The current study supports urgency for more research and suggests a set of self-report measures that could be useful.

Notably, 85% of participants knew someone who recently died of overdose in the past year. This level of mortality is consistent with overdose statistics, but is a striking when examined on a community level. In addition to ending the overdose epidemic, grief support is also needed [24]. One of the primary predictors of fatal overdose is a prior non-fatal overdose [25]. Understanding non-fatal overdose patterns and predictors can help to inform intervention strategies that successfully prevent non-fatal overdoses from becoming fatalities; and identify opportunities to prevent overdose occurrence in the first place. There are several efforts underway to collate and maximize the information available about non-fatal overdoses to inform naloxone distribution and other public health planning [26]. There is also an emerging body of harm reduction messaging in response to xylazine, such as emphasizing rescue breathing as a complement to naloxone in overdose response and treating xylazine-related wounds early in order to prevent greater severity. These results reinforce the need for timely actionable data and the value of community-based research to complement administrative and decedent data sources.

## Conclusion

These data underscore the urgency of identifying subgroups at highest risk of encountering xylazine and experiencing related harms; and ensuring harm reduction and health care availability.

## Data Availability

The dataset analyzed during the current study are available from the corresponding author on reasonable request.
